# Personalized Treatment for Scalp Angiosarcoma

**DOI:** 10.3390/jcm14041278

**Published:** 2025-02-14

**Authors:** Adriana Nicoleta Cristescu, Ioana Dumitrescu, Irina Tudose, Cristina Beiu, Ana-Maria Dumitrescu, Liliana Gabriela Popa

**Affiliations:** 1 Dermatology Department, Elias Emergency University Hospital, 17 Marasti Bd., District 1, 011461 Bucharest, Romania; 2 General Surgery Department, Prof. Dr. Dimitrie Gerota Emergency Hospital, 29-31 Vasile Vasilievici Stroescu Street, District 2, 021374 Bucharest, Romania; 3 Histopathology Department, Elias Emergency University Hospital, 17 Marasti Bd., District 1, 011461 Bucharest, Romania; 4 Dermatology Department, Carol Davila University of Medicine and Pharmacy, 37 Dionisie Lupu Street, District 1, 020021 Bucharest, Romania; 5 D-Clinic, 2B Avrameni Street, District 1, 013738 Bucharest, Romania

**Keywords:** angiosarcoma, scalp, surgery, radiotherapy

## Abstract

Cutaneous angiosarcoma is a rare and aggressive malignant tumor that originates from the endothelial cells of blood vessels or lymphatic vessels. More than half of cutaneous angiosarcoma cases occur in the head and neck regions, particularly on the scalp. However, due to its rarity, scalp angiosarcoma is often overlooked in clinical practice. The lack of distinctive symptoms usually delays the diagnosis and, implicitly, the initiation of appropriate treatment. The treatment of cutaneous angiosarcoma poses great challenges due to its multifocal occurrence and the frequent extensive microscopic spread. A personalized, multimodal therapeutic approach is essential for ensuring a favorable outcome, consisting of a wide surgical excision associated with adjuvant radiotherapy in localized tumors, concurrent adjuvant radiotherapy and chemotherapy, targeted treatments, or immunotherapy in advanced or metastatic diseases. The aim of this manuscript is to review the literature data regarding the individualized management of cutaneous angiosarcoma and to share our own experiences in the field. We wish to underscore the importance of considering cutaneous angiosarcoma in the differential diagnosis of scalp tumors, especially in patients with a history of scalp irradiation as early detection, accurate diagnosis, and a multidisciplinary, personalized management, including surgery with clear margins and adjuvant radiation therapy, are crucial for ensuring a favorable outcome.

## 1. Introduction

Angiosarcoma is an uncommon and aggressive vascular malignancy, classified into four subtypes, including classic angiosarcoma of the scalp, face, and neck, also referred to as Wilson–Jones angiosarcoma, lymphedema-associated angiosarcoma (LAS), also known as Stewart–Treves syndrome, which develops in patients with chronic lymphedema, usually post-cancer surgery, radiation-induced angiosarcoma (RIA), and aggressive epithelioid angiosarcoma [1,2].

Cutaneous angiosarcoma represents about 1–2% of all soft tissue sarcomas, with more than half of the cases occurring in the head and neck region, particularly on the scalp, which is the most common site [3,4], ranking third among primary scalp malignant tumors [5]. This condition more frequently affects older individuals with fair skin, with a male to female ratio of approximately 3:1 [6,7].

While the exact cause of scalp angiosarcoma is often unclear, several risk factors have been identified. These include a history of radiation exposure, chronic lymphedema, trauma, advanced age (most commonly affecting individuals in their 70s), and exposure to ultraviolet radiation or certain chemicals, such as arsenic, thorium dioxide, polyvinyl chloride, and radium [4,6,8,9,10]. RIA represents a late complication of radiotherapy, arising in the irradiated area years, even decades, after radiation treatment [11]. However, many cases arise without any known predisposing factors [12].

Angiosarcoma originates in the endothelium of blood/lymphatic vessels [13,14]. The serum levels of endothelial cell proliferation and angiogenesis regulators, such as vascular endothelial growth factor (VEGF)-D and angiopoietin-2 are markedly increased in patients with angiosarcoma, being directly proportional to the extent of the disease [15,16]. On the other hand, vascular endothelial cadherin, an important component of endothelial adherens junctions, is absent in angiosarcomas and their metastases, which leads to tissue invasion and metastases [17]. An increased density of stem cell factor (SCF)-expressing mast cells was detected in angiosarcomas, promoting neovascularization and cellular proliferation [18]. The expression of Fas ligand (Fas-L), a potent inducer of apoptosis, was demonstrated in more than 70% of angiosarcomas, showing, as expected, an inverse correlation with tumor-infiltrating lymphocytes (TILs) and survival [14]. Contrarily, angiosarcoma cells evade apoptosis, mainly due to increased expression of carcinogenesis drivers such as phosphatidylinositol-4, 5-bisphosphate 3-kinase catalytic subunit alpha (PIK3CA), phosphorylated mitogen-activated kinase-like protein (pMAPK), and tumor protein p53 (TP53), and decreased expression of phosphatase and tensin homolog (PTEN), a major regulator of the PI3K/Akt pathway [19].

Recent studies have provided insights into angiosarcoma’s molecular landscape, revealing several genetic alterations that have significant implications for the condition’s diagnosis, prognosis, and potential therapeutic strategies [20]. The TP53 gene, known for its role in tumor suppression, is mutated in approximately 29% of angiosarcoma cases. These mutations are more prevalent in angiosarcomas of the head and neck region, with a reported frequency of 50% [20]. Mutations in the POT1 gene, which is involved in telomere maintenance and normally contribute to cellular aging and apoptosis, occur in about 16% of angiosarcomas. Notably, in head and neck angiosarcomas, the frequency of POT1 mutations increases to 40.5% [20]. Amplification of the MYC gene is observed in approximately 23% of angiosarcoma cases. Studies have shown that MYC amplification is almost exclusively found in radiation-related angiosarcoma. MYC proteins regulate transcription and DNA replication and also contribute to cell cycle progression. Novel approaches targeting the cell cycle through CDK inhibition have demonstrated encouraging outcomes in tumors with MYC amplification [20,21].

Angiosarcoma portends a very poor prognosis, with a 5-year survival rate of 26–38% [6,22,23], a recurrence rate that exceeds 50%, and an estimated metastatic rate of 29% [22]. Ages over 70 years, dimensions greater than 5 cm, and location on the head or neck are associated with a worse prognosis [24,25]. The latter might be due to the particularities of scalp vascularization, with emissary veins directly connected to the intracranial space, allowing the direct spread of the malignant cells to the brain [26]. Contrarily, the scarce vascular supply in the irradiated area is believed to confer RIA a better prognosis. Complete surgical excision with wide margins is also associated with improved outcomes [26]. This, however, can only be accomplished in small, promptly diagnosed lesions as scalp angiosarcoma is characterized by clinically undetectable extensions and in more than half of cases the tumor is multifocal, displaying a multinodular or diffuse micronodular growth pattern [27]. Positive margins also delay radiation therapy if additional surgery is planned, worsening the patient’s prognosis even further [28].

**Figure 1:**
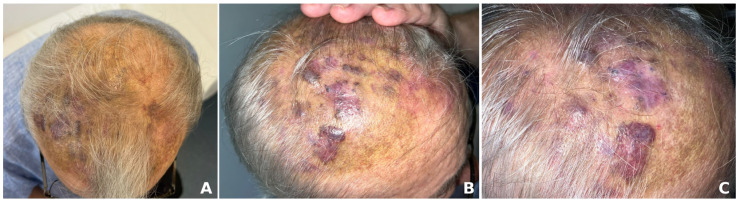
Poorly demarcated erythematous-violaceous plaque (**A**), with yellow-greenish areas with irregular margins (**B**), infiltrated in places with violaceous nodules on the surface affecting the fronto-parietal scalp region (**C**).

Clinically, angiosarcomas appear as bluish or red-purple nodules, plaques, or ecchymosis-like lesions, which can easily be mistaken for benign conditions like hematomas or hemangiomas (Figure 1) [4,6]. Sometimes, the tumors may bleed or ulcerate. Therefore, they can be incorrectly identified as trauma-induced lesions [3,4]. Angiosarcomas are typically asymptomatic. However, they can occasionally present mild pain or pruritus [29,30]. The tumors vary in size and are usually multifocal, infiltrating surrounding tissues and making it difficult to determine clear boundaries for surgical removal. They often progress rapidly and metastasize to regional lymph nodes or spread hematogenously to distant organs, especially to the lungs and liver [3,12].

Due to its rarity, scalp angiosarcoma is often overlooked in clinical practice. The lack of distinctive symptoms can delay the diagnosis and, implicitly, the initiation of appropriate treatment.

Clinically, scalp angiosarcoma is difficult to differentiate from post-traumatic lesions (ecchymosis, hematoma), infections (cellulitis/erysipelas), inflammatory skin diseases (sebborheic dermatitis, psoriasis, lupus tumidus), malignant solid tumors (squamous cell carcinoma, amelanotic melanoma, leiomyosarcoma, skin metastasis), and cutaneous lymphomas (Table 1) [31,32]. A history of recent local trauma, the presence of pain, and the typical evolution from an initial red-blue cutaneous discoloration to a greenish and eventually yellowish hue over several days as hemoglobin converts to biliverdin and biliverdin subsequently converts to bilirubin, are evocative of post-traumatic ecchymosis/hematomas. Skin infections, such as cellulitis and erysipelas, are characterized by an acute onset of erythema, edema, increased local temperature, and tenderness. Chronic inflammatory skin diseases that commonly affect the scalp, like seborrheic dermatitis and psoriasis, manifest as erythema and scaling and may be accompanied by distinctive skin lesions elsewhere on the patient’s body. Scalp angiosarcoma may closely mimic malignant solid tumors and cutaneous lymphomas. Nevertheless, whenever a suspicion of cutaneous malignancy is raised, a skin biopsy is promptly performed, enabling histopathologic differentiation of clinically similar neoplasms. Dermoscopy and imagistic procedures may aid in the early diagnosis.

**Figure 2:**
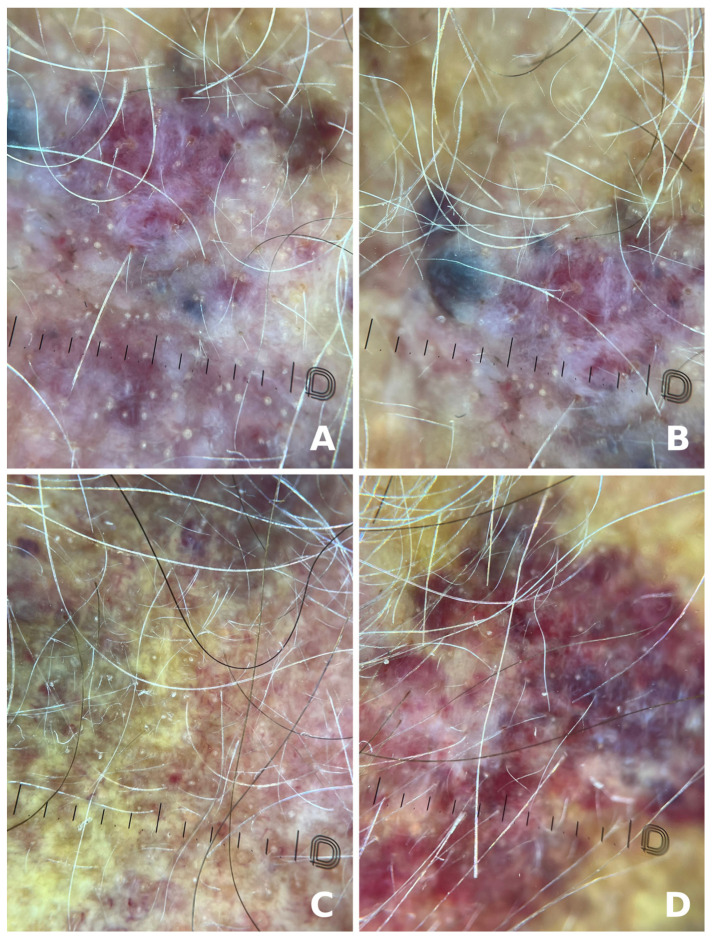
Dermoscopic aspect of the tumor, showing yellow, red, and purple structureless areas (**A**,**B**) with yellow clods (**C**), occasionally covered with interlaced white lines, dark purple and blue nodules (**D**).

Although the dermoscopic features of this malignant skin tumor have been described by very few studies, the usefulness of dermoscopy in establishing early diagnosis is unquestionable. Zalaudek et al. analyzed the dermoscopic patterns of scalp and face angiosarcoma and concluded that patchy, structureless areas of different red, purple and blue hues, interlaced with small, round/oval, yellow clods typical for skin pores are constant dermoscopic features. Nodular areas within the tumors are dermoscopically characterized by dark purple zones with white lines (Figure 2) [31]. Other authors reported similar dermoscopic findings [33,34]. The dermoscopic features closely parallel the histopathologic findings in these dermal vascular tumors with dark reddish to purplish areas, indicating secondary dilated vessels, organizing thrombi and hemorrhages, dark purple nodules corresponding to malignant cell masses, and white lines to fibrous septa [31]. Oiso et al. underlined that the most prominent and distinguishing feature of scalp angiosarcoma is represented by various color gradations, describing “pink-purplish stream-like areas with a white or skin-coloured central area, and a strengthening of the purple colour at the periphery of the lesions” [34]. This finding differentiates angiosarcoma from purpura and ecchymosis. Additionally, Minagawa et al. highlighted the absence of vascular structures, i.e., vessels and lacunae upon dermoscopic examination of scalp angiosarcoma lesions, which the authors attributed to the nonuniform proliferation of malignant cells, with little luminal differentiation [35]. This is also a very helpful clue, aiding in the differential diagnosis with other vascular tumors, amelanotic melanoma, or inflammatory skin diseases, such as psoriasis. However, polymorphous atypical vessel-like structures may be present, representing telangiectasia and vascular channels [34], similar to those described in cutaneous carcinoma lymphatic metastases [36].

RIA, on the other hand, displays particular dermoscopic features, including homogeneous whitish-pink structureless areas related to the post-irradiation fibrosis, loosely distributed purple globules, and peripheral color intensification [31]. De Giorgi et al. also studied the dermoscopic features of RIA and described a characteristic steam-like area [33].

Imaging studies, such as computed tomography (CT) and magnetic resonance imaging (MRI), are also essential for assessing the extent of the malignancy and checking for potential metastases [3,4]. Radiologic investigations may also help in the differential diagnosis of scalp angiosarcomas from benign lesions and malignant tumors. Moreover, the extension of the tumor is more precisely established based on MR images, optimizing the therapeutic approach [37,38]. The well-contrast-enhanced MR images and prolonged T1- and T2- relaxation times reflect the tumor’s hypervascularity [37]. Kawaguchi et al. assessed unenhanced MR and ^18^F-FDG–positron emission tomography (PET)/CT images of scalp angiosarcoma and asserted that these tumors invariably present as flat elevated lesions with subcutaneous adipose tissue invasion [39]. In addition, a third of the studied cases presented multiple lesions [39]. Given the presence of intratumoral hemorrhages and hemosiderin deposition, scalp angiosarcomas displayed intra-tumoral hypointensity, as well as mixed hyper- and hypointensity on T2-weighted images noticed in the majority of cases, significantly more often than in squamous cell carcinomas [39]. These findings also differentiate scalp angiosarcomas from cutaneous lymphomas, melanomas, cutaneous metastases, and neurofibromatosis. In cutaneous lymphomas, the lesions are isointense on T1- and T2-weighted images due to high cellularity. Melanomas are hypo- to hyperintense on T1- and T2- weighted images, depending on the amount of melanin contained by the tumor and the severity of intratumoral hemorrhage. Neurofibromatosis and cutaneous metastases show intratumoral hypointensity on T2-weighted images [40]. Comb-like or reticular hypointense patterns are also observed on unenhanced T1-weighted images [37]. The scalp, galea aponeurotica, and occipito-frontal muscles are thickened in all cases and show abnormal signal intensities [37]. Scalp angiosarcomas are also characterized by high-flow serpentine vessels with low signal intensity on T1- and T2- weighted images [41], as well as low-flow vessels with hyperintensity on T2-weighted images [40].

**Figure 3:**
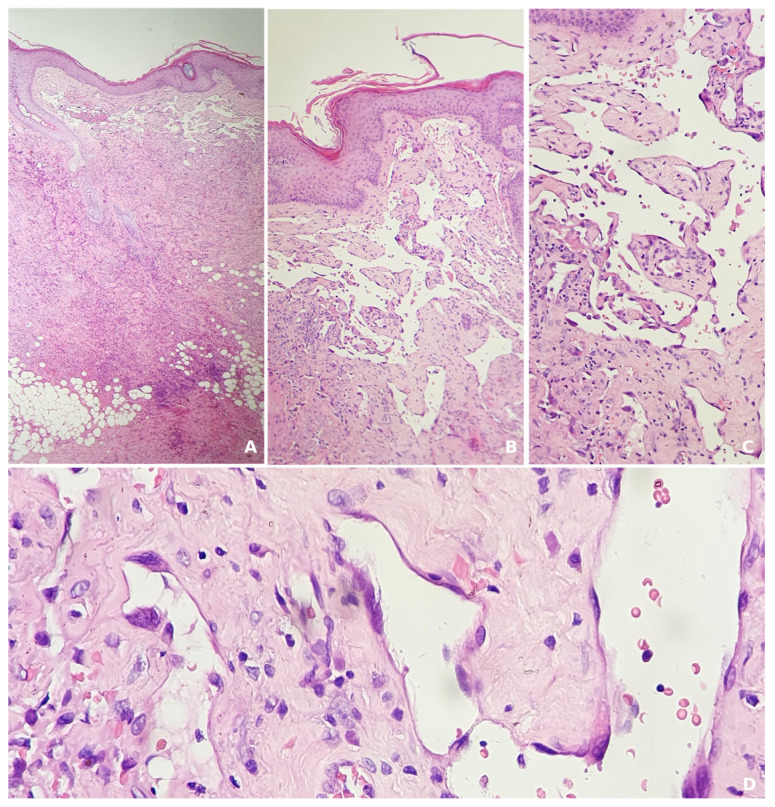
Hematoxylin-eosin stain showing a tumor with sclero-hyaline stroma that includes atypical spindle-shaped/elongated cells infiltrating focally among adipose lobules ((**A**), 40×), formation of papillary structures with a fibrous core lined by pleomorphic endothelial cells, ((**B**), 100×), and atypical vascular proliferation composed of irregularly dilated and anastomosed vascular spaces, lined by pleomorphic endothelial cells ((**C**), 200× and (**D**), 400×).

**Figure 4:**
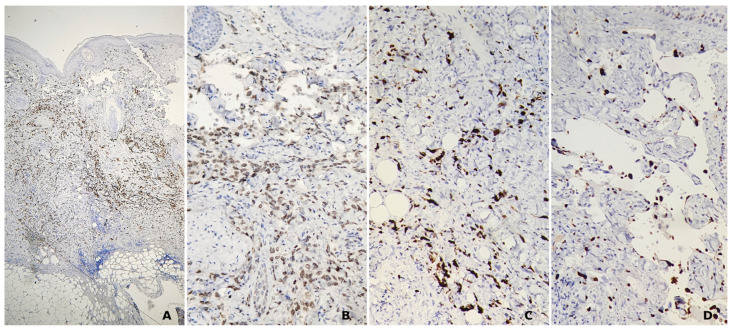
Immunohistochemical stains showing positivity for ERG ((**A**), 40×) and FLI1 ((**B**), 200×), as well as positivity for Ki67 in approximately 40% of tumor cells nuclei ((**C**,**D**) 200×).

A skin biopsy is the gold standard for diagnosis, as it provides a definitive histopathological diagnosis. Histologically, angiosarcomas can range from well-differentiated to poorly differentiated types. In well-differentiated tumors, vascular channels are lined by a single layer of atypical endothelial cells anastomose between collagen fibers (Figure 3). On the other hand, poorly differentiated tumors are characterized by the accumulation of pleomorphic cells with limited signs of vascular differentiation. Therefore, immunohistochemistry (IHC) is necessary to confirm the tumor’s endothelial origin, with markers like CD31, CD34, FLI1, and ERG being particularly useful for identifying vascular differentiation (Figure 4) [4,12].

Fluorescence in situ hybridization (FISH) is a useful technique for detecting MYC gene amplification in angiosarcoma samples. This method employs fluorescent probes that bind specifically to the MYC gene locus, allowing for the visualization of gene amplification under a fluorescence microscope. The detection of MYC amplification through FISH can aid in distinguishing angiosarcoma from other vascular lesions, particularly in challenging cases [42]. IHC is also commonly used to assess MYC protein expression in tissue samples. Overexpression of MYC protein correlates with MYC gene amplification, which is associated with more aggressive tumor behavior and may suggest a poorer prognosis [21,42].

Additionally, the Ki-67 proliferation index, determined by IHC, serves as a marker of cellular proliferation. A high Ki-67 index indicates increased proliferative activity, which can help in differentiating between benign and malignant lesions that share histologic similarities. This is especially useful in distinguishing conditions with overlapping features, such as hemangioma and low-grade angiosarcoma [42,43].

Moreover, IHC analysis of angiosarcomas reveals a complex tumor microenvironment marked by significant CD8+ T-cell infiltration. These cytotoxic lymphocytes indicate an active anti-tumor response and have been associated with improved clinical outcomes. The presence of high levels of CD8+ infiltration not only serves as a prognostic marker but also suggests potential responsiveness to immune checkpoint inhibitors, such as programmed death (PD)-1 inhibitors (pembrolizumab, nivolumab) and cyotoxic T lymphocyte associated protein (CTLA)-4 inhibitors (ipilimumab), which may further enhance anti-tumor immunity [44,45].

Understanding these molecular alterations is crucial for the accurate diagnosis and management of angiosarcoma. Furthermore, identifying specific genetic alterations can lead to new strategies using targeted therapies and personalized treatment approaches [20,21].

The histopathological examination is indispensable for a definite diagnosis. However, the histopathological differential diagnosis may sometimes be challenging (Table 1). Scalp angiosarcoma should be differentiated from atypical fibroxanthoma, characterized by an admixture of spindle cells, histiocyte-like cells and multinucleated giant cells with marked cytological atypia but without a vascular differentiation. Hemangiomas, with their well-demarcated lobular architecture and well-established vascular channels lined by benign endothelial cells are more easily distinguishable. Kaposi sarcoma is another clinical and histological differential diagnosis, but its characteristic features and positive stains for human herpesvirus (HHV) 8 aid in establishing the correct diagnosis. Epithelioid hemangioendothelioma should be considered in the differential diagnosis of angiosarcoma. It is composed of round polygonal cells, with eosinophilic cytoplasm and vesicular nuclei arranged in short cords or nests, surrounded by hyaline and myxoid stroma. Retiform hemangioendothelioma also resembles well-differentiated angiosarcoma, with slit-like spaces lined by hobnail endothelial cells, but no significant cytological atypia. Kaposiform hemangioendothelioma consists of fascicles of spindle-shaped endothelial cells, congested capillaries, slit-like vascular spaces in lobular and infiltrative patterns and stains negative for HHV8 [46]. As the histopathologic picture of angiosarcomas varies greatly, from well differentiated forms to cases with marked anaplasia, differentiation from melanomas or carcinomas may prove problematic in some cases.

**Table 1:** The main differential diagnoses of scalp angiosarcomas [46].

Differential Diagnosis	Characteristic Features
Posttraumatic lesions (ecchymosis, hematoma)	A history of recent local trauma, painful red-blue cutaneous discoloration that evolves to a greenish and eventually yellowish hue
Hemangiomas	Macules, papules, plaques, nodules, or tumors of various colors, from bright red to purple, blue or skin-color, with a well-demarcated lobular architecture on histopathology and well-established vascular channels lined by benign endothelial cells.
Infections (cellulitis/erysipelas)	Acute onset of erythema, edema, increased local temperature, and tenderness.
Chronic inflammatory dermatoses (sebborheic dermatitis, psoriasis)	Scalp lesions characterized by erythema and scalling may be accompanied by distinctive skin lesions elsewhere on the patient’s body.
Lupus tumidus	Erythemato-edematous plaques with normal overlying epidermis, with dermal mucin deposition, a lymphocytic inflammatory infiltrate and edema on histopathologic examination.
Cutaneous lymphomas	Dark red/brown macules, plaques or tumors that may ulcerate, histologically characterized by dermal and epidermal infiltration with atypical lymphocytes with variable morphology depending on the disease subtype
Atypical fibroxanthoma	Solitary, slow-growing red/pink papule or firm nodule, histologically characterized by an admixture of spindle cells, histiocyte-like cells and multinucleated giant cells with marked cytological atypia
Kaposi sarcoma	Purple, red, brown, or blue macules, plaques, and/or nodules that may ulcerate and bleed, with a characteristic histopathological picture, with variate intensity of angiοproliferation, dermal inflammatory infiltrate, and spindle cell bundles depending on the stage of the disease and positive stains for HHV8
Epithelioid hemangioendothelioma	Painful cutaneous mass histologically characterized by round, polygonal cells with eosinophilic cytoplasm and vesicular nuclei arranged in short cords or nests, surrounded by hyaline and myxoid stroma
Retiform hemangioendothelioma	Plaque-like discoloration that histologically displays slit-like spaces lined by hobnail endothelial cells, but no significant cytological atypia
Kaposiform hemangioendothelioma	Purple, slightly elevated subcutaneous mass with occasional telangiectasias, which shows fascicles of spindle-shaped endothelial cells, congested capillaries, slit-like vascular spaces in lobular and infiltrative patterns on histopathological examination and stains negative for HHV8

No staging system currently exists for cutaneous angiosarcoma. Treating scalp angiosarcoma is challenging due to its aggressive and recurrent nature. To date, no prospective randomized clinical trials regarding the optimal treatment in head and neck angiosarcomas have been performed given their rarity. A personalized, multimodal approach is advocated by the studies carried out so far, which highlighted the increase of overall survival and relapse free survival in patients undergoing radiotherapy after wide surgical excision of the tumor (Table 2) [6,27,47]. The standard approach for localized tumors is surgical resection with wide margins to ensure the complete removal of the tumor, and is usually followed by skin grafting [48], although this may lead to significant disfigurement for patients. However, achieving clear margins is often difficult, especially in multifocal or infiltrative cases. Even with complete surgical excision, recurrence rates are high. Both well and poorly differentiated angiosarcomas are characterized by extensive local spread, their margins exceeding the clinically visible ones [49]. Mohs micrographic surgery does not provide additional benefits in scalp angiosarcoma as it has been demonstrated that intraoperative frozen sections are not as accurate as paraffin sections in determining the microscopic extent of the tumor [49,50]. Therefore, in cases in which wide surgical excision with histologically free margins is not possible, postoperative radiotherapy is essential to prevent recurrence [49]. Radiotherapy is commonly used after surgery to improve local control or in cases where complete surgical resection is not possible or contraindicated [3,8]. Preoperative radiotherapy has also been performed in a series of patients, but its utility is debatable [51,52,53].

The role of chemotherapy as the primary adjuvant therapy is controversial, with some studies failing to prove its benefit [49]. On the other hand, several authors support the use of taxanes, such as paclitaxel and docetaxel, in association with postoperative irradiation reporting improved metastasis free survival rates due to the antiangiogenic and radiosensitizing properties of these chemotherapeutic agents [49,54,55]. Most experts reserve chemotherapeutic regimens based on taxanes or doxorubicin as salvage therapy in patients with recurrent disease or metastatic tumors [49,56,57,58]. Nevertheless, the lack of a standardized chemotherapy regimen can make treatment decisions challenging, and response rates to chemotherapy can vary significantly.

New therapies, including targeted treatments and immunotherapy, are being investigated in clinical trials, but their role in treating scalp angiosarcoma is still not fully established [8,12]. Antiangiogenic agents, such as VEGF inhibitors, sorafenib, and bevacizumab have not proven efficient [27]. Gene therapy has also been recently employed in patients with angiosarcoma in the form of cDNA, with interferon-2b being injected intralesionally which led not only to complete regression of the treated lesions, but also to partial regression of a noninjected lesion [49]. Personalization of the therapeutic approach is essential for ensuring a favorable outcome (Table 2).

**Table 2:** Treatment options for scalp angiosarcomas.

Treatment	Results
Wide surgical excision	Represents the gold standard for the treatment of scalp angiosarcomas May lead to significant disfigurement High recurrence rates due to multifocal or infiltrative disease [49]
Mohs micrographic surgery	No additional benefit compared to classic surgery [49,50]
Adjuvant radiotherapy	Improves local control of the disease Reports of increased overall and relapse free survival [6,27,47]
Preoperative radiotherapy	Benefit not proven [51,52,53]
Radiotherapy	Therapeutic alternative in patients in whom surgery is contraindicated [3,8]
Chemotherapy as primary adjuvant therapy	Controversial, benefit not proven [49]
Adjuvant concurrent radiotherapy and taxane-based chemotherapy	Reports of improved metastasis free survival [49,54,55]
Salvage therapy with taxane or doxorubicin-based chemotherapy	Reserved for patients with recurrent or metastatic disease [49,56,57,58]
Antiangiogenic targeted therapy with VEGF inhibitors, sorafenib and bevacizumab	Benefit not proven [27]
Immunotherapy	The benefit of immunotherapy in angiosarcoma is being investigated [8,12]
Gene therapy using cDNA for interferon-2b injected intralesionally	Complete regression of the treated lesions and partial regression of noninjected lesions [49]

We wished to present our own experience, illustrated by the case of a 72-year-old male patient who presented for a giant scalp angiosarcoma. The patient first noticed the skin lesion a year previously and sought medical advice. He was diagnosed with seborrheic dermatitis and was recommended treatment with topical corticosteroids and antifungals. The lesion gradually enlarged, measuring 26/19 cm at present in our clinic (Figure 1). His medical history included childhood radiotherapy for tinea capitis, arterial hypertension, nephrolithiasis, venous insufficiency, for which he was undergoing oral antihypertensive, and venotonic treament. Dermoscopy showed a skin lesion composed of yellow, red, and purple structureless areas with yellow clods, occasionally covered with interlaced white lines, dark purple and blue nodules, and imprecise borders (Figure 2). An incisional skin biopsy was performed, the histopathologic examination and immunohistochemistry studies confirming the clinical suspicion of angiosarcoma. The histopathologic examination showed an atypical vascular proliferation located in the dermis extending into the adipose tissue, consisting of irregularly dilated, anastomosing vascular spaces lined by pleomorphic endothelial cells with occasional papillary structures with a fibrous axis that was also lined by pleomorphic endothelial cells. No mitosis was observed. The tumor’s sclero-hyaline stroma included atypical fusiform/elongated cells which focally infiltrated the adipose tissue. Erythrocyte extravasation, small hemosiderin deposits, and a minimal intratumoral polymorphic inflammatory infiltrate were also present (Figure 3). The tumor stained positive for CD31+, CD34+, ERG+, FLI1+ and negative for HHV 8. Ki67 was positive in approximately 40% of the tumor cells nuclei (Figure 4). The laboratory tests included a complete blood count, metabolic panel, coagulation tests and tumor markers and all the results were within normal limits. Cerebral and cervical MRI and thoracic, abdominal and pelvic CT scans showed no cranial bone invasion, malignant lymphadenopathy, systemic metastasis, or other pathologic findings.

**Figure 5:**
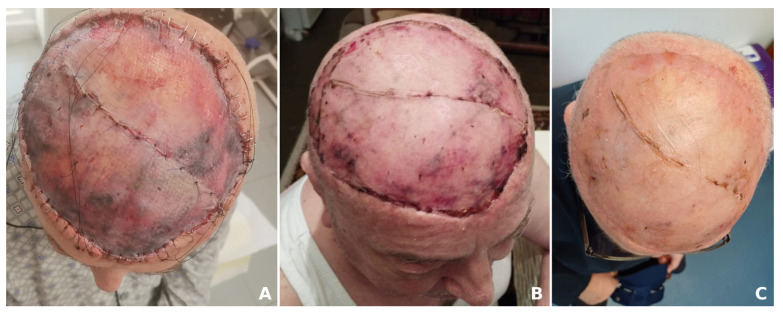
Post-operative aspect 5 days (**A**), 2 weeks (**B**) and 6 weeks (**C**) after the surgical intervention.

The patient underwent wide surgical resection, with the defect covered by a free split-thickness skin graft (Figure 5). The histopathologic examination established the diagnosis of well-differentiated angiosarcoma (G1) and confirmed clear margins. Subsequently, adjuvant radiotherapy was administered. The patient remained under close medical observation with no recurrences 6 months after surgery.

In conclusion, this case underscores the importance of considering cutaneous angiosarcoma in the differential diagnosis of scalp skin tumors, especially in patients with a history of scalp irradiation. The treatment of cutaneous angiosarcoma poses great challenges due to its multicentric occurrence and the frequent extensive microscopic spread. Therefore, early detection, accurate diagnosis, and a multidisciplinary, personalized management, including surgery with clear margins and adjuvant radiation therapy, are crucial for a favorable outcome.
